# Direct Evidence on the Contribution of a Missense Mutation in *GDF9* to Variation in Ovulation Rate of Finnsheep

**DOI:** 10.1371/journal.pone.0095251

**Published:** 2014-04-21

**Authors:** Michael P. Mullen, James P. Hanrahan

**Affiliations:** Teagasc, Animal & Grassland Research and Innovation Centre, Mellows Campus, Athenry, Co. Galway, Ireland; University of Nevada School of Medicine, United States of America

## Abstract

The Finnish Landrace (Finnsheep) is a well known high-prolificacy sheep breed and has been used in many countries as a source of genetic material to increase fecundity of local breeds. Analyses to date have indicated that mutations with a large effect on ovulation rate are not responsible for the exceptional prolificacy of Finnsheep. The objectives of this study were to ascertain if: 1) any of 12 known mutations with large effects on ovulation rate in sheep, or 2) any other DNA sequence variants within the candidate genes *GDF9* and *BMP15* are implicated in the high prolificacy of the Finnish Landrace breed; using material from lines developed by divergent selection on ovulation rate. Genotyping results showed that none of 12 known mutations (*Fec*B*^B^, Fec*X*^B^, Fec*X*^G^*, *Fec*X*^GR^, Fec*X*^H^, Fec*X*^I^*, *Fec*X*^L^, Fec*X*^O^, Fec*X*^R^, Fec*G*^E^, Fec*G*^H^,* or *Fec*G*^T^*) were present in a sample of 108 Finnsheep and, thus, do not contribute to the exceptional prolificacy of the breed. However, DNA sequence analysis of *GDF9* identified a previously known mutation, V371M, whose frequency differed significantly (P<0.001) between High and Low ovulation rate lines. While analysis of ovulation rate data for Finnsheep failed to establish a significant association between this trait and V371M, analysis of data on Belclare sheep revealed a significant association between V371M and ovulation rate (P<0.01). Ewes that were heterozygous for V371M exhibited increased ovulation rate (+0.17, s.e. 0.080; P<0.05) compared to wild type and the effect was non-additive (ovulation rate of heterozygotes was significantly lower (P<0.01) than the mean of the homozygotes). This finding brings to 13 the number of mutations that have large effects on ovulation rate in sheep and to 5, including *FecB^B^, FecG^E^*, *Fec*X*^O^* and *Fec*X*^GR^*, the number of mutations within the TGFβ superfamily with a positive effect on prolificacy in the homozygous state.

## Introduction

It is well established that both *BMP15* and *GDF9* play central roles in normal ovarian development and function in mammals, and that mutations in these genes or in their receptors can cause large increases in ovulation rate of sheep [1]–[3]. Since the demonstration that the exceptional prolificacy of the Booroola Merino was attributable to the effect of a single gene [4], mutations with a major effect on litter size and ovulation rate (OR) have been invoked to explain the exceptional prolificacy observed in many sheep populations. In some of these populations the causative mutations have been identified, including the Booroola Merino [*Fec*B*^B^*] [5], Romney [*Fec*X*^I^* and *Fec*X*^H^*] [6], Cambridge [*Fec*X*^G^* and *Fec*G*^H^*] and Belclare [*Fec*X*^G^*, *Fec*X*^B^* and *Fec*G*^H^*] [7], Lacaune [*Fec*X*^L^*] [8], Icelandic sheep [*Fec*G*^T^*] [9], Santa Ine∧s (*Fec*G*^E^*) [10], Rasa Aragonesa *(Fec*X*^R^*) [11], and Olkuska (*Fec*X*^O^*) and Grivette (*Fec*X*^GR^*) [12].

Ewes that are homozygous for any of eight of these mutations (*Fec*X*^G^*, *Fec*X*^B^, Fec*X*^I^*, *Fec*X*^H^*, *Fec*X*^L^*, *Fec*X*^R^* in *BMP15,* or *Fec*G*^H^*, *Fec*G*^T^* in *GDF9*) are sterile due to arrested follicle development [6]–[9], [11].

The Finnish Landrace (Finnsheep) is a well known high-prolificacy sheep breed and the heritability of ovulation rate in the breed has been shown to be of the order of 0.5 [13]. Finnsheep have been used worldwide to increase prolificacy usually by crossing with local breeds so as to reduce the proportion of Finn ancestry as there were often unfavourable effects on other production traits [14]. The Finnsheep used in the present study were descendants of 41 ewes and 23 rams, imported from Finland to Ireland in 1966, that were selected on the basis of their own or their mothers’ litter size [13]. Subsequent selection on ovulation rate was used to develop High and Low lines, leading to a divergence of between 2 and 2.5 ova, depending on age; a Control line was also maintained [13], [15].

While Finnsheep have been used in many countries to increase the fecundity of local breeds [17] no direct evidence has been adduced for the involvement of mutations with a large effect on prolificacy [18]. The objectives of this study were to: 1) ascertain if any of 12 known mutations with large effects on ovulation rate in sheep contribute to the exceptionally high prolificacy of the Finnish Landrace breed using material from lines developed by divergent selection on ovulation rate, and 2) re-sequence the coding regions of candidate genes *BMP15* and *GDF9* to identify if any other variants may be involved in Finnsheep prolificacy.

## Results and Discussion

None of 12 mutations, across *BMP15, GDF9* and *BMPR1B*, known to be associated with large effects on ovulation rate in sheep were present in the Finnsheep tested, in agreement with the findings of Davis et al. [18] where the Booroola mutation and *Fec*X*^I^* were shown to be absent from a smaller sample of High and Control line Finnsheep. Thus, the large divergence in ovulation rate generated between the High and Low selection lines was not due to any of these known mutations and, by extension, none of these mutations are responsible for the exceptional prolificacy of Finnsheep.

Sequence analysis revealed no mutations in the coding regions of *BMP15* in Finnsheep. However, V371M, a mutation in *GDF9* (Figure S1) previously reported as G7 [7], was identified and the frequencies (Table 1) differed significantly among the lines (P<0.001). Although the within-line association between this mutation and ovulation rate in Finnsheep was not statistically significant, the fact that the High line was homozygous for the mutation while it was at a very low frequency in the Low line and at an intermediate frequency in the unselected Control line strongly suggested that this mutation was associated with a relatively large effect on ovulation rate. The pooled estimate for the effect of one copy of V371M, using contrasts from both Control and Low lines, was 0.28 (s.e. 0.281; P = 0.33; Table 2). As additional material was not available for the Finnish Landrace lines, the Belclare breed, whose development involved planned incorporation of genetic material from the High Finn line [19], was identified as source of animals with a frequency of the V371M mutation that would provide additional evidence on the effect of V371M on ovulation rate. The frequency of the V371M mutation in a set of 181 Belclare ewes used in the study was 0.17 (Table 1). A total of 167 of the 181 Belclare ewes used were confirmed as not carrying any of the 12 mutations with large effects on ovulation rate in sheep, via DNA sequence analysis of the complete coding regions of *BMP15* and *GDF9*, and RFLP analysis of the Booroola locus. The remaining 14 ewes were heterozygous for either *Fec*X*^G^* (n = 10) or *Fec*X*^B^* (n = 4); the presence of these heterozygotes was not unexpected since these mutations in *BMP15* were known to be present in the Belclare breed [7]. Analysis of the ovulation rate data on the Belclare ewes showed that there was a significant association (P<0.001) between V371M and ovulation rate (Table 3). Evaluation of the differences among the genotypes, based on the data for ewes that were wild type at the *BMP15* locus, showed that the effect of allele substitution was not additive (P<0.01); the difference between wild type and heterozygote was 0.17 (s.e. 0.080; P = 0.035) compared with a difference of 1.46 (s.e. 0.380; P<0.001) between the heterozygous and homozygous individuals (Table 3). Unfortunately, the small number of homozygous ewes available (n = 2) means that the precision of the estimate of the effect of homozygosity for V371M is low. When analysis was confined to the adult-ewe records the heterozygous ewes had an ovulation rate that was greater (+0.20, s.e. 0.091; P = 0.025) than that of wild-type ewes (Table 3). Thus, ewe age had a minor influence on the estimate of the effect of the mutation. The non-additive effect on ovulation rate contrasts with the essentially additive effect of the *Fec*G*^E^* mutation [10] on this trait when OR is estimated [16] from the data on litter size (estimated mean OR values are 1.17, 1.61 and 2.14 for wild type, heterozygote and homozygote, respectively). While analysis of litter size failed to identify any statistically significant differences among the genotypes the estimate for the effect of a single copy of the V371M mutation on this trait was +0.13 (s.e. 0.098; P = 0.19; Table 3). The magnitude of this effect is consistent with the corresponding difference in ovulation rate when account is taken of the curvilinear relationship between litter size and ovulation rate in sheep [16]. This effect is only about one half of that reported for the *Fec*G*^E^* mutation in Santa Inês sheep [10].

**Table 1:** Incidence of genotypes and estimates of gene frequency for Finnish Landrace and Belclare populations.

Breed	Selection line	Genotype			Frequency of *Fec*G*^F^*
		(+/+)	(*Fec*G^F^/+)	(*Fec*G^F^/*Fec*G^F^)	
Finnish Landrace	High	0 (0)†	0 (0)	32 (12)	1.00
	Control§	21 (9)	22 (14)	3 (2)	0.30 (0.22–0.39)‡
	Low	29 (11)	1 (0)	0 (0)	0.017 (0–0.042)
Belclare	N/A	120 (0)	58 (0)	3 (0)	0.18 (0.14–0.21)

† Number of males.

‡ 95% confidence interval from 10 000 bootstrap samples.

§ The observed frequency of the genotypes in the Control line was consistent with Hardy-Weinberg expectation.

**Table 2:** Least squares estimates (± s.e.) of mean ovulation rate for Finnish Landrace lines by genotype.

Selection line	No. of records	Genotype		
		+/+	*Fec*G*^F^*/+	*Fec*G*^F^*/*Fec*G*^F^*
High	80 (20)†	−	−	4.45±0.189
Control	79 (21)	2.33±0.172	2.48±0.215	2.98±0.592
Low	69 (19)	1.85±0.140	2.23±0.493	−

† No. of ewes.

**Table 3:** Least squares estimates (± s.e.) of mean ovulation rate and litter size for genotype subclasses of Belclare sheep.

Genotype		Ovulation rate			Litter size	
*Fec*G*^F^*	*BMP15*	No. of ewes‡	All records	Excluding ewe lambs	No. of ewes	All records
Wild type	++	108	2.53±0.049	2.76±0.056	104	2.16±0.062
Heterozygote	++	57	2.70±0.067	2.96±0.077	55	2.29±0.083
Homozygote	++	2	4.00±0.374	4.52±0.447	2	2.85±0.782
Wild type	Heterozygote	12	3.90±0.140	4.33±0.162	12	2.69±0.178
Heterozygote	Heterozygote	1	4.19±0.487	4.63±0.556	1	2.47±0.620
Homozygote	Heterozygote	1	4.46±0.474	4.89±0.524	1	2.27±0.518
Estimate for *Fec*G*^F^* effect: Heterozygote – Wild type		1122†	0.17±0.080 (P = 0.035)	0.20±0.091 (P = 0.025)	456†	0.12±0.097 (P = 0.22)

‡ The numbers for ewes with records as adults were 104, 55, 2, 12, 1, 1, respectively, and total records was 809.

† Total number of records.

The phenomenon of ovarian hypoplasia, and consequent sterility, has not been observed in the present Finnsheep lines nor are we aware of any reports of such an occurrence in Finnsheep elsewhere. Therefore, the gene variants that contributed to the response to selection for ovulation rate in the Finnsheep lines, including V371M, were not detrimental to the crucial role played by the growth factor encoded by *GDF9* in normal ovarian development [20], [21]. Mutations with large effects on ovulation rate but with no detrimental effect on fertility in the homozygous state have been reported for the Booroola (*Fec*B*^B^*) [5], Santa Inês (*Fec*G*^E^*)[10], and Olkuska (*Fec*X*^O^*) and Grivette (*Fec*X*^GR^*) sheep [12]. The present report is the first of such an occurrence in Finnsheep. It should be noted that a region on chromosome 11 in Lacaune sheep has also been shown to harbour a mutation (*Fec*L*^L^*) with a similar pattern of effects but the mutation responsible has not been identified [22], [23].

The precise molecular effects of V371M on *GDF9* have not been established. However, both *GDF9* and *BMP15* are members of the TGFβ family which encodes prepropeptides containing a signal peptide, a proregion and a C-terminal mature region that is the biologically active peptide. Most members of the TGFβ superfamily are biologically active as dimers and, although *GDF9* and *BMP15* do not contain the cysteine molecule responsible for covalent inter-chain disulphide bonding seen in other members of the family, these molecules are thought to be biologically active as dimers [8], [24] and heterodimers [25]. It is not known whether the physiologically active dimers are homodimers or heterodimers, or whether all three possible dimer forms play a role [25]. A potential mechanism for the phenotypic effects of many variants of *BMP15* and *GDF9* may lie in the lack of the cysteine residue responsible for the inter-chain disulphide bond that forms a covalent link between monomers in most members of the TGFβ superfamily. This may reduce or abolish biological activity by predisposing *BMP15* or *GDF9* to dimer instability in the presence of any mutations that impair hydrogen bonding, and thus dimerization. This hypothesis is consistent with the bioinformatic analysis of the mutation V371M using several algorithms, including Polyphen-2, SIFT, I-Mutant, MUpro and SCpred; all analyses classified this substitution as damaging and predicted a role for this amino acid position as a stabilization centre and that the substitution of valine by methionine at position 371 would reduce protein stability (data not shown).

The biological mechanisms by which mutations in *GDF9* exert an effect on ovulation rate in sheep are yet to be clearly elucidated. Suggested mechanisms for the effects of mutations in *BMP15* involve diminished/eliminated capacity of individuals heterozygous/homozygous for the *BMP15* mutant to reduce the sensitivity of granulosa cells (GC) to follicle stimulating hormone (FSH) resulting in the maintenance, selection and ovulation of numerous smaller follicles [20]. In rats, *GDF9* has been shown to inhibit FSH-induced cAMP synthesis [26]; therefore, the effect of mutations in *GDF9* on the sheep ovary may be to increase the sensitivity of the ovarian follicle to FSH and thereby increase the ovulation rate as proposed for individuals heterozygous for *BMP15* mutations. In light of the results from the current study, this would suggest that V371M diminishes the biological activity of the GDF9 mature protein, reducing its capacity to function as a feedback inhibitor but without abolishing its essential role in normal reproductive development in sheep.

Previous analyses of the variation in ovulation rate within the Finn lines used in the present study and the observed response to selection led to the conclusion that a gene with a large effect on ovulation rate was not involved [15]. However, the possibility that the High line was homozygous for a mutation with a ‘large effect’ on ovulation rate was not considered. The present findings clearly require that the previous conclusion be rejected and it is concluded that V371M was responsible for a portion of the response reported previously [15]. The paucity of comparative data on the effect of homozygosity for V371M on ovulation rate means that the actual contribution of this mutation to the response in ovulation rate is uncertain. However, the present analysis clearly indicates that the increase in ovulation associated with homozygosity for V371M is significantly greater than would be predicted based on the effect of a single copy of this mutation. Unfortunately, the precision of the estimate of the difference between wild type and homozygote is low (95% confidence interval for the true effect of homozygosity is 0.9 to 2.6). If the true value is closer to the lower bound, and taken as ∼1.25, the consequences for the variability and repeatability of ovulation rate in Finnsheep would not be substantial. However, simulation analysis (details not reported) shows that the coefficient of variation for ovulation rate in the Control line would be greater than that in the Low line; there was no evidence for such a pattern in the results previously reported by Hanrahan [15]. This may be taken to indicate that, while the gene effect is non additive, the estimate of 1.46 is likely to represent an overestimate of the consequences of homozygosity for ovulation rate. An upper bound to the effect of homozygosity on OR can be estimated from the difference between the High and Control lines, the difference in gene frequency and the estimate for the effect of a single copy of the V371M gene variant. The resulting estimate is 1.9; a true effect of this magnitude would imply that essentially all of the divergence between the selection lines can be attributed to the change in the frequency of V371M. However, this conclusion is not consistent with the pattern of response observed in the High line; the evidence shows that while there was a large initial divergence between the High and Control lines this was followed by a steady annual increase over the subsequent 10 years of the selection experiment [15]. In light of all of the above it seems safer to conclude that the V371M variant accounts for only a portion of the observed response to selection for ovulation rate in Finnsheep. Further evidence on the effect of homozygosity on OR is required before a more precise estimate can be offered for the contribution of this mutation.

Since the work reported above was originally prepared for publication Våge et al. [27] reported on the effects of V371M in Norwegian White Sheep (NWS), using data from a progeny test evaluation involving a large number of rams. The trait evaluated was litter size and the estimate for the effect of the mutation in the heterozygous state was +0.25 (excluding litter size data on ewe lambs). This estimate is about twice that observed for the effect of V371M on litter size of Belclare ewes in the present study. Since the litter size of adult ewes of the Norwegian White Sheep breed is approximately 2.1 [28] the reported effect on litter size is equivalent to an effect of the order of +0.5 on ovulation rate, given the curvilinear relationship between these variables [16]. Finnish Landrace genetic material has been incorporated into the NWS population and the implication of the information reported by Våge et al. [26] is that the V371M mutation in NWS came from Finnsheep. This is consistent with ram progeny-test results for litter size of Norwegian sheep [29]; the distribution of individual ram effects presented for a set of 47 ½-Finn rams exhibits significantly greater variability than that for a panel of 605 Norwegian rams evaluated under the same system. The estimate of the effect of the mutation in NWS [26] is not consistent with the results from either Finnsheep or the Belclare breed even though the original source of the mutation is most likely the same. Clearly, additional evidence is required on the effects of this mutation on ovulation rate, particularly the ovulation rate of homozygous ewes, including possible interactions with the genetic background. The V371M mutation is also present in Cambridge sheep [7], [30] but absent from the Lleyn [30] breed. The presence of V371M in Cambridge sheep is presumably a reflection of the significant contribution of the Finnsheep to the ancestry of this breed.

The argument could be made that the effect observed in V371M heterozygotes is due to linkage with an unknown causative gene on chromosome 5; this cannot be ruled out without functional studies. However, we propose that this is not the case for several reasons: 1) the established functional role of *GDF9* in ovarian function in sheep and other species; 2) the predicted negative effects of V371M on protein function and stability, especially in light of its biological activity in dimer form, coupled with the inherent susceptibility of this member of the TGFβ family to dimer instability due to the lack of critical cysteine residues; 3) the overall consistency for the effect of V371M on ovulation rate in both the Finnish Landrace and Belclare ewes; and 4) the results reported by Våge et al. [27] for NWS. It is proposed that this SNP be designated *Fec*G*^F^* (*^F^*, Finnsheep) based on the current nomenclature for mutations affecting fertility in sheep.

Studies on the effects of mutations in candidate genes such as *BMP15* or *GDF9* have greatly increased our understanding of the impact of ‘major genes’ on phenotypes such as fecundity. The use of model organisms to dissect the genetic components underpinning complex traits is of relevance to human health given the close parallels to human regulation of biological processes. While differences do exist for the effects of *BMP15* or *GDF9* mutations in sheep and humans, human *BMP15* mutations also affect ovarian function. However, instead of increasing ovulation rate, as observed in sheep, they are associated with ovarian dysgenesis or premature ovarian failure (POF) [31]. *BMP15* mutations in humans occur in the proregion, which is known to drive dimerisation and secretion of the active mature dimers, and results in the production of aberrant dimers [32]. *GDF9* expression in women with polycystic ovary syndrome (PCOS) have shown reduced and delayed expression, implicating *GDF9* as a contributor to PCOS [33]. Subsequent studies have shown significant association of *GDF9* variants, including two missense mutations in the *GDF9* proregion, with the POF phenotype [34] in humans.

In terms of genetic improvement, mutations with moderate effects (e.g., *Fec*G*^E^, Fec*G*^F^*) on ovulation rate in sheep may well be more desirable than mutations with effects of the magnitude of *Fec*B*^B^* and *Fec*G*^H^* due to the negative side effects of large litters, such as increased perinatal mortality and reduced growth rate, in addition to the extensive genotyping required to manage the segregation of a gene with such effects. The identification of *Fec*G*^F^* in Finnish Landrace and Belclare sheep has implications for genetic evaluation programmes given the relatively high frequency of the mutation (0.17 to 0.3) in both breeds (Tables 3) and the use of Finnsheep worldwide as a source of genetic material to increase fecundity of local breeds [17].

## Materials and Methods

### Animals

#### Ethics statement

Animal handling, laparoscopy for the determination of ovulation rate and blood collection were conducted under license from the Irish Department of Health in accordance with Irish and European Union legislation (Cruelty to Animals Act, 1876, and European Community Directive, 86/609/EC). All animals were managed in accordance with the guidelines for the accommodation and care of animals under Article 5 of the Directive.

#### Finnsheep

Three lines of Finnish Landrace sheep were developed over the period 1976 to 1997 by divergent selection on ovulation rate [15]. The mean (s.e.) ovulation rate at 18 months of age was 4.6 (0.05), 2.1 (0.06) and 2.6 (0.05) for High, Low and Control lines, respectively, for animals born between 1993 and 1997, inclusive. DNA was available for 124 animals born in 1994 and 1995 and represented 37 High, 38 Low and 49 Control line animals.

#### Belclare sheep

A total of 181 Belclare ewes, born between 2004 and 2007, and observations on ovulation rate as ewe lambs and as adults were chosen for study from the research flock at Athenry. Litter size data were also available for these cohorts at 2 to 5 years of age.

### Ovulation Rate

Ovulation rate was determined by mid-ventral laparoscopy at around the middle of the oestrous cycle for two consecutive cycles in each breeding season. Finnsheep were measured as ewe lambs and at 18 months of age. The Belclare ewes were measured as ewe lambs and at 18, 30 and 42 months of age.

### DNA Extraction

DNA was extracted from the whole blood samples and FTA cards as described previously [7], [35].

### Genotyping

#### PCR forced Restriction Fragment Length Polymorphism (PCR-RFLP)

The 124 Finn samples had been screened previously for *Fec*X*^G^*, *Fec*X*^B^* and *Fec*G*^H^* [30] and subsequently, as part of the current study, for *Fec*B*^B^, Fec*X*^I^* and *Fec*X*^H^* using PCR-RFLP as described previously [6], [7], [18]. In addition, the 181 Belclare animals were also screened for *Fec*B*^B^* by PCR-RFLP. The digested fragments were separated on a 4% agarose gel and visualized with ethidium bromide staining. The gels were scored for the presence or absence of the mutations. Homozygous, heterozygous and negative controls were included within each assay.

#### iPLEXMassArray genotyping

The 124 Finnsheep samples were regenotyped, using the Sequenom MassArray iPLEX Gold assay (Eurofins-Medigenomix, Ebersburg, Germany), for 7 of the known (at the time) mutations associated with ovulation rate in sheep, viz., *Fec*X*^G^*, *Fec*X*^B^*, *Fec*G*^H^*, *Fec*X*^I^*, *Fec*X*^H^*, *Fec*B*^B^* and *Fec*X*^L^*.

#### Resequencing

Finnish Landrace (n = 108) and Belcare (n = 181) sheep DNA samples were successfully resequenced across the full coding regions of both *BMP15* and *GDF9* (Eurofins-Medigenomix, Ebersburg, Germany) to: 1) confirm the genotypes assigned by the Sequenom MassArray iPLEX Gold assay, 2) screen for additional mutations at these loci (*Fec*X*^GR^, Fec*X*^O^, Fec*X*^R^, Fec*G*^E^, Fec*G*^T^*) recently reported to have effects on ovarian function in sheep, and 3) identify any novel variants within these genes. Thus, the animals were screened for the presence of 12 of the mutations currently known to alter ovarian function in sheep.

### Statistics

Analysis of ovulation rate data employed least squares procedures (Proc MIXED; SAS Institute, Cary, NC) to fit models with fixed effects for ewe age, year of record, oestrous cycle and genotype, with selection line included in the case of the Finnsheep data; ewe identity was included as a random term in all analyses. Effects of allele substitution were evaluated using orthogonal contrasts to estimate the additive and non-additive components. The data sets used in the current study are available as supporting information in.csv formats (Table S1 (Belclare data) and Table S2 (Finn data)).

### Bioinformatic Analysis

The potential ‘pathogenicity’ of V371M for the biological function of GDF9 was evaluated using prediction methods: Polyphen2 [36] and SIFT [37], and predicted effects on protein stability were evaluated using: I-Mutant 2.0 [38]; MUpro [39] and SCPRED [40].

## Supporting Information

Figure S1 **DNA sequence analysis showing a: wild type (A), heterozygous (B) and homozygous (C) animal at the** ***GDF9*** **V371M (** ***Fec*** **G** ***^F^*** **) locus (base 251) in Finnish Landrace ewes.**
(TIF)

Table S1 **Belclare sheep data.**
(CSV)

Table S2 **Finnsheep data.**
(CSV)
