# Supplementary figures and images for: Direct Evidence on the Contribution of a Missense Mutation in GDF9 to Variation in Ovulation Rate of Finnsheep
Source: PLoS One. 2014 Apr 21;9(4):e95251. doi: 10.1371/journal.pone.0095251 (PMC3994038; doi:10.1371/journal.pone.0095251)

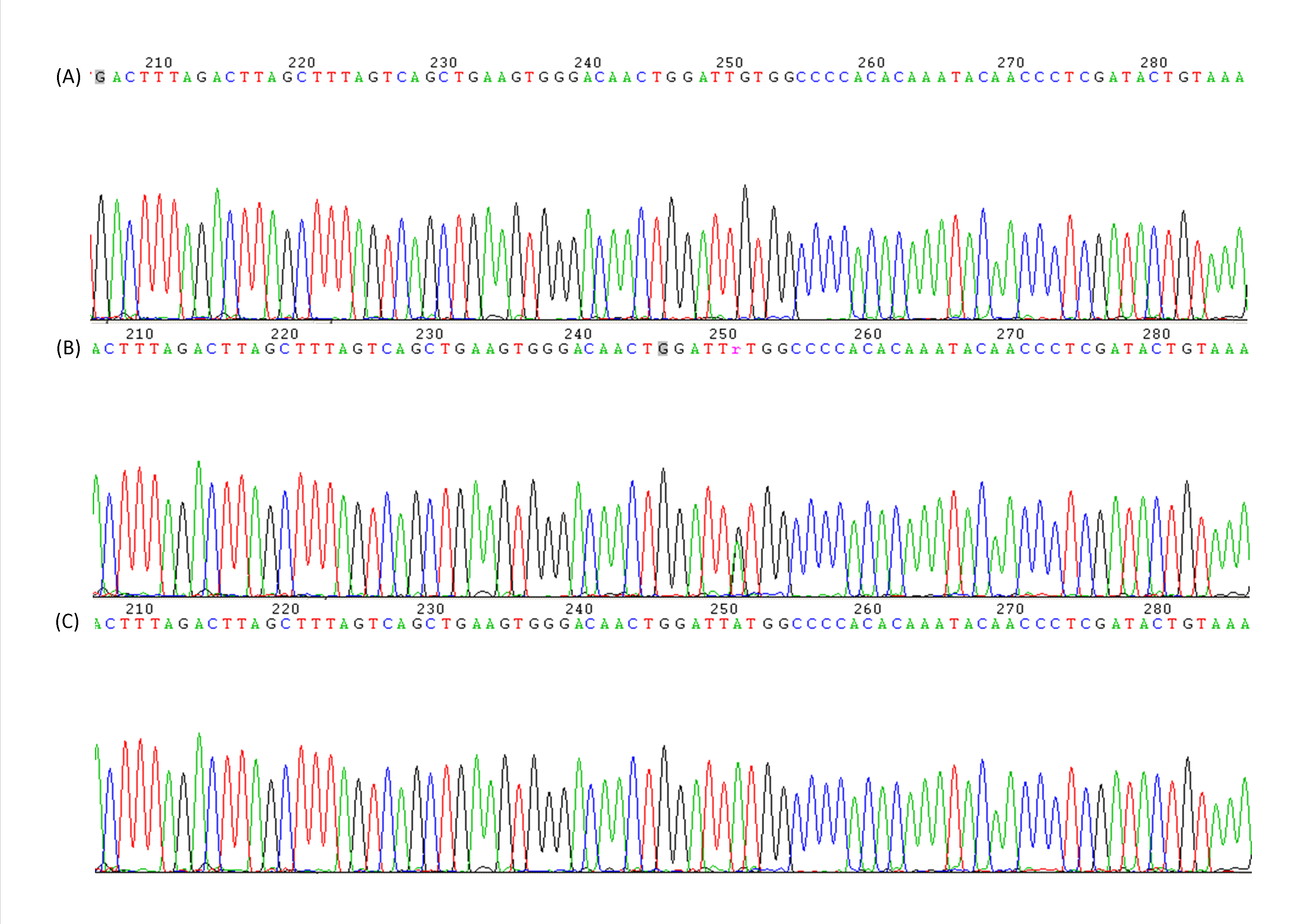

Supplement: Figure S1 — DNA sequence analysis showing a: wild type (A), heterozygous (B) and homozygous (C) animal at the GDF9 V371M ( Fec G F ) locus (base 251) in Finnish Landrace ewes. (TIF) [file pone.0095251.s001.tif]
